# Use of Androcoll-E^TM^ to Separate Frozen-Thawed Llama Sperm From Seminal Plasma and Diluent

**DOI:** 10.3389/fvets.2020.594926

**Published:** 2021-01-21

**Authors:** Crissthel Yverlin Guillén Palomino, Fernanda Gabriela Fumuso, Mariana Lucía Bertuzzi, Susana María Giuliano, Nicolás Velásquez González, Maria Victoria Bariani, María Ignacia Carretero

**Affiliations:** ^1^Laboratorio de Biotecnología Reproductiva, Estación Experimental Agraria Canaán, Instituto Nacional de Innovación Agraria (INIA-Ayacucho), La Molina, Perú; ^2^Cátedra de Teriogenología, Facultad de Ciencias Veterinarias, Instituto de Investigación y Tecnología en Reproducción Animal, Universidad de Buenos Aires, Buenos Aires, Argentina; ^3^Consejo Nacional de Investigaciones Científicas y Técnicas (CONICET), Buenos Aires, Argentina

**Keywords:** Androcoll, colloid, cryopreservation, diluent, llama, semen, seminal plasma

## Abstract

It is not easy to separate frozen-thawed South American camelid sperm from seminal plasma (SP) and diluents to be used for *in vitro* embryo production. The objective of this study was to evaluate Androcoll-E™ (AE) efficiency to separate llama sperm from SP and freezing extender in frozen-thawed semen. A total of 22 ejaculates from five *Lama glama* males were collected using electroejaculation. After performing semen analysis (sperm motility, concentration, viability, membrane function, and acrosome integrity), samples were cryopreserved with a diluent containing lactose, ethylenediaminetetraacetic acid (EDTA), egg yolk, and 7% dimethylformamide. After thawing, samples were divided in aliquots, one of which was used as a control and the others processed by AE. *Experiment 1* (12 ejaculates): 100 μl of frozen-thawed semen was placed on top of 1,000 μl AE column and centrifuged at 800 *g* for 10 min. *Experiment 2* (10 ejaculates): two samples of 100 μl of frozen-thawed semen were placed on two columns of 500 μl AE each, and both were centrifuged at 800 *g* for 10 and 20 min, respectively. Pellets were resuspended in Tyrode's albumin lactate pyruvate (TALP) medium, and sperm parameters were evaluated. A significant decrease in all sperm parameters was observed in thawed samples compared to raw semen. AE allowed the separation of frozen-thawed sperm from SP and freezing extender independently from the height of the column used and time of centrifugation assayed. Although no significant differences were found between AE columns, higher sperm recovery was observed with 500 μl of AE coupled with 20 min of centrifugation. Despite the significant decrease observed in sperm motility in AE samples, no changes in sperm viability, membrane function, and acrosome integrity were observed when comparing control thawed semen with the sperm recovered after AE (*p* > 0.05). The use of AE columns, either 500 or 1,000 μl, allows the separation of frozen-thawed llama sperm from SP and freezing extender, preserving the viability, membrane function, and acrosome integrity. Of the protocols studied, 800 *g* centrifugation during 20 min using a 500 μl column of AE would be the method of choice to process frozen-thawed llama semen destined for reproductive biotechnologies.

## Introduction

It is well-known that South American camelid (SAC) semen presents special rheological characteristics such as high structural viscosity (1) and the capacity to form a thread when handled (2). Furthermore, ejaculated sperm of these species have a particular motility pattern, they possess oscillatory motility with practically no progressive motility (2–4). Due to these characteristics, several *in vitro* SAC embryo production studies have reported the use of sperm recovered from animals with deviated deferent ducts or collected from the epididymis after death or castration, since those samples are free of seminal plasma (SP) and have sperm with progressive motility (5–13). Moreover, the absence of high viscosity and thread formation are advantages when processing these SP-free samples. However, protocols designed for epididymal sperm will not necessarily have similar outcomes when applied in raw semen samples that have SP, and the surgical intervention performed to deviate the deferent ducts renders the animal useless for future breeding.

Also, SAC embryos have been obtained *in vitro* using spermatozoa from enzymatically treated ejaculates subsequently processed with colloids such as Percoll® or Androcoll-E™ (AE) (14, 15). Although enzymatic treatment reduces thread formation of camelid semen and facilitates manipulation of samples, the effect that it could induce in sperm membrane components is still unknown. In addition, it has been suggested that Percoll® could be potentially dangerous to sperm cells (16). Thus, it is recommended to wash and centrifuge sperm after using this colloid, consequently increasing the time required for preparation of samples (17) and producing cell damage due to the rise in production of reactive oxygen species (18).

The use of frozen-thawed semen for *in vitro* embryo production in llamas is a very interesting tool, since it would allow the use of sperm at a later date and thus become independent from the day of sample collection, permitting a better planning of *in vitro* fertilization (IVF) timing. Additionally, it would allow the use of ejaculates from males of high genetic value that are no longer available to work with or even after their death. Moreover, survival of SAC cryopreserved sperm is very low, obtaining very poor pregnancy rates when artificial insemination (AI) is performed with frozen-thawed semen in these species (19–22). The results have been related to the rheological characteristics previously mentioned, which prevent accurate homogenization of ejaculates with the diluents and probably affecting proper penetration of cryoprotectants across sperm cell membranes. However, cryopreservation of llama sperm in the absence of SP does not improve sperm survival (23, 24). Considering all these facts, selection of suitable frozen-thawed sperm from samples where the majority are dead could be an alternative to SAC. In other species, greater percentages of motile, viable, and morphologically normal sperm with intact acrosomes and intact DNA have been reported in frozen-thawed samples treated with Androcoll [horse: (25, 26); dog: (27, 28); donkey: (29, 30); brown bear: (31)]. Recently, AE allowed the separation of llama sperm from SP in raw semen samples, avoiding the use of enzymatic treatments (32). However, this colloid has not yet been used with llama frozen-thawed semen samples. In this context, the objective of this study was to evaluate the efficiency of AE to separate llama frozen-thawed sperm from SP and the freezing extender.

## Materials and Methods

### Reagents

Propidium iodide (PI), 6-carboxyfluorescein diacetate, dimethyl sulfoxide, and the reagents for the Tyrode's albumin lactate pyruvate (TALP) medium and for the hypoosmotic swelling (HOS) test were purchased from Sigma Chemicals (Sigma Aldrich, Buenos Aires, Argentina). Coomassie Blue (CB) was purchased from Bio-Rad, California, United States.

### Animals and Location

The study was carried out at the Faculty of Veterinary Sciences of the University of Buenos Aires in Buenos Aires, Argentina. The city is situated at sea level, latitude 34°36′ and longitude 58°26′.

For the study, five male *Lama glama* ranging between 7 and 11 years of age and weighing 142.6 ± 19.2 kg (mean ± SD) were used. Animals were kept out at pasture in pens and supplemented with bales of alfalfa; they also had free access to fresh water throughout the study. All males were shorn during the month of November.

### Experimental Design

Semen collections were carried out between April and October using electroejaculation (EE) under general anesthesia according to the technique described by Director et al. (33). The frequency of collection for each male was determined randomly. As EE requires general anesthesia, this method was not used on the same male at an interval of <15 days. All procedures were approved by the Committee for the Use and Care of Laboratory Animals (CICUAL) of the Faculty of Veterinary Sciences of the University of Buenos Aires (protocol 2019/22).

A total of 22 ejaculates from five adult llama males were processed. After the evaluation of semen characteristics, samples were cryopreserved with a diluent containing lactose, ethylenediaminetetraacetic acid (EDTA), egg yolk, and 7% of dimethylformamide. Briefly, ejaculates were diluted to a final concentration of 40 × 10^6^ spermatozoa/ml or, if initial concentration was lower, a 1:1 (semen:extender) dilution was carried out. Then, samples were equilibrated for 20 min at room temperature, loaded into 0.25-ml straws and frozen using a manual method (34). Briefly, temperature descent was carried out in three phases by placing the straws, together with a digital thermometer, submerged in a mixture of ethanol:acetone (1:1) in a bronze canister with a graduated handle and holding over liquid nitrogen vapors inside a 10-L nitrogen tank. Temperature phases were as follows: (i) from room temperature to −15°C (temperature descent at a rate of 10°C−12°C min^−1^), (ii) from −15°C to −120°C at a rate of 25°C−40°C min^−1^, and (iii) the straws were plunged into liquid nitrogen at −196°C. The samples were thawed in a water bath at 37°C for 60 s. After thawing, semen samples were divided into aliquots, one of which was used as a control (unprocessed sample) and the others were processed through AE according to the experiment. *Experiment 1* (12 ejaculates): 100 μl of frozen-thawed semen was placed on top of 1,000 μl AE column and centrifuged at 800 *g* for 10 min. *Experiment 2* (10 ejaculates): two samples of 100 μl of frozen-thawed semen were placed on top of two columns of 500 μl AE each, and both were centrifuged at 800 *g* for 10 and 20 min, respectively. In both experiments, after centrifugation through AE, pellets were resuspended in TALP medium and the sperm were evaluated.

### Sperm Evaluations

Each ejaculate was evaluated for sperm quality before freezing (raw semen), after thawing (frozen-thawed semen control), and after processing by AE (frozen-thawed AE samples).

Volume of the ejaculates was evaluated using a micropipette.

Thread formation was evaluated indirectly according to the capacity of the samples to form a thread when 20 μl of the sample was pipetted onto a slide using a micropipette. This parameter was classified as either present or absent.

Sperm motility was evaluated using a phase contrast microscope (100×) and a warm stage (37°C). The patterns observed were oscillatory motility and progressive motility. In addition, total sperm motility was calculated (total motility = oscillatory + progressive).

Sperm concentration was calculated using a Neubauer hemocytometer chamber.

The 6-carboxyfluorescein diacetate (CFDA) and PI stains were used for assessing membrane integrity (viability) according to Giuliano et al. (35). Briefly, samples (12.5 μl) were incubated at 37°C for 10 min in 127 μl of staining medium. This medium contained 2 μl of a solution of CFDA (0.5 mg ml^−1^ in dimethyl sulfoxide) and 125 μl of saline medium [described by Harrison and Vickers (36)]. After the first 10 min of incubation, 2 μl of a solution of PI (0.5 mg ml^−1^ in isotonic saline) was added and the samples were incubated for another 10 min at 37°C. A minimum of 200 spermatozoa were evaluated per sample using an epifluorescence microscope (400×) (Leica®, 134 DMLS, Heerbrugg, Switzerland). Spermatozoa that fluoresced green throughout their length were classified as being viable (intact membrane), while sperm nuclei that fluoresced red were classified as non-viable (damaged membrane).

The HOS test in combination with CB staining, according to Carretero et al. (37), was performed to evaluate sperm membrane function and acrosome integrity. Briefly, for the HOS test, 12.5 μl of samples were incubated at 37°C for 20 min in 50 μl of a hypoosmotic solution containing fructose and sodium citrate (50 mOsml l^−1^). After the incubation, 62.5 μl of 4% paraformaldehyde in PBS was added and samples were incubated for 4 min at room temperature, with subsequent centrifugation at 800 *g* for 10 min. Pellets were then resuspended in 100 μl of PBS. Next, small drops of the sample were placed on microscope slides, within previously marked wells using a hydrophobic barrier pen, and were left to dry at room temperature. Samples were stained with 0.22% CB for 5 min and were examined using an optical microscope at 1,000× magnification. Sperm cells that showed the characteristic tail swelling were classified as HOS positive (HOS+), while sperm cells without tail swelling were classified as HOS negative (HOS-). At the same time, sperm cells were classified according to CB acrosome staining as follows: purple-stained acrosomes (CB+; acrosome present) or absence of acrosome staining (CB-; acrosome absent). Finally, 200 spermatozoa from each sample were analyzed and classified into the following categories: (1) sperm with functional plasma membranes and presence of acrosomes (HOS+/CB+), (2) sperm with functional plasma membranes and without acrosomes (HOS+/CB–), (3) sperm with non-functional plasma membranes and presence of acrosomes (HOS–/CB+), and (4) sperm with non-functional plasma membranes and without acrosomes (HOS–/CB–).

### Statistical Analysis

Statistical analyses were performed using InfoStat software (Student Version) (https://www.infostat.com.ar/index.php?mod=page&id=15). In all cases, normal distribution and homogeneity of variances of the data were corroborated using the Shapiro–Wilk's normality test and a Bartlett's test, respectively. The level of significance was set at 0.05 for all analyses.

To compare raw and frozen-thawed semen, a paired Student *t*-test was used to assess sperm viability, membrane function, and acrosome integrity; because sperm motility did not show a normal distribution, this was analyzed by Wilcoxon test.

To compare frozen-thawed semen (control) and frozen-thawed samples processed by AE, a paired Student *t*-test (experiment 1) and a factorial design with a Tukey test (experiment 2) were used to evaluate sperm concentration, viability, membrane function, and acrosome integrity. Because sperm motility did not show a normal distribution, these data were analyzed by a Wilcoxon test (experiment 1) and a Kruskal–Wallis test (experiment 2).

## Results

### Raw Semen vs. Frozen-Thawed Semen (Control)

The volume of the ejaculates was 1.2 ± 0.6 ml (mean ± SD). Thread formation was observed in 15 of the 22 ejaculates that were collected (68.2%), which was maintained in nine of the 22 frozen-thawed semen samples (40.9%).

In both experiments (1 and 2), a significant decrease (*p* < 0.05) in all sperm parameters, except progressive motility, was observed in frozen-thawed semen samples (untreated controls) compared to raw semen (Table 1 for experiment 1 and Table 2 for experiment 2).

**Table 1:** Sperm parameters evaluated in raw semen and frozen-thawed (FT) llama semen (untreated control) in ejaculates used for experiment 1.

**Sperm (%)**	**Raw semen**	**FT semen (control)**
Oscillatory motility	51.3 ± 8.6^a^	7.3 ± 7.2^b^
Progressive motility	2.7 ± 4.6^a^	1.1 ± 2.6^a^
Total motility	54.0 ± 13.0^a^	8.4 ± 6.6^b^
Viability	69.2 ± 11.2^a^	13.5 ± 9.5^b^
HOS+CB+	60.0 ± 10.5^a^	14.2 ± 4.8^b^

*Values are expressed as means ± SD (n = 12 ejaculates). HOS+CB+: percentage of sperm positive to the hypoosmotic swelling test (HOS+) with an acrosome present (CB+)*.

a,b *For each sperm parameter, different letters between columns indicate significant differences (p < 0.05)*.

**Table 2:** Sperm parameters evaluated in raw semen and frozen-thawed (FT) llama semen (untreated control) in ejaculates used for experiment 2.

**Sperm (%)**	**Raw semen**	**FT semen (control)**
Oscillatory motility	41.3 ± 19.7^a^	6.1 ± 6.0^b^
Progressive motility	3.1 ± 4.5^a^	3.0 ± 3.8^a^
Total motility	44.4 ± 19.1^a^	9.1 ± 5.8^b^
Viability	51.6 ± 16.8^a^	17.3 ± 6.8^b^
HOS+CB+	44.7 ± 11.9^a^	22.3 ± 8.9^b^

*Values are expressed as means ± SD (n = 10 ejaculates). HOS+CB+: percentage of sperm positive to the hypoosmotic swelling test (HOS+) with an acrosome present (CB+)*.

a,b *For each sperm parameter, different letters between columns indicate significant differences (p < 0.05)*.

### Frozen-Thawed Semen (Control) vs. Frozen-Thawed Samples Processed by Androcoll-E^TM^

The use of AE allowed separation of frozen-thawed llama sperm from SP and from the freezing extender independently of the height of AE column (500 and 1,000 μl) and time of centrifugation used (10 and 20 min.). Although it is not possible to statistically compare experiments 1 and 2, the highest sperm recovery was observed in samples processed with 500 μl AE columns combined with 20 min of centrifugation at 800 *g* (Tables 3, 4).

**Table 3:** Sperm concentration, motility (oscillatory, progressive, and total), viability, and spermatozoa with functional membranes and an acrosome present (HOS+CB+) in frozen-thawed (FT) llama semen (untreated control) and FT samples centrifuged 20 min through 1,000 μl of Androcoll-E™ columns (FT-AE).

	**FT semen (control)**	**FT-AE (1,000 μl)**
Concentration (×10^6^ sperm/ml)	30.0 ± 6.1^a^	4.4 ± 9.5^b^
Oscillatory motility (%)	7.3 ± 7.2^a^	1.4 ± 2.0^b^
Progressive motility (%)	1.1 ± 2.6^a^	0.4 ± 1.0^a^
Total motility (%)	8.4 ± 6.6^a^	1.8 ± 2.1^b^
Viability (%)	13.5 ± 9.5^a^	12.1 ± 10.0^a^
HOS+/CB+	14.2 ± 4.8^a^	11.0 ± 5.5^a^

*Values are expressed as means ± SD (Experiment 1, n = 12 ejaculates)*.

a,b *For each sperm parameter, different letters between columns indicate significant differences (p < 0.05)*.

**Table 4:** Sperm concentration, motility (oscillatory, progressive, and total), viability, and spermatozoa with functional membranes and an acrosome present (HOS+CB+) in frozen-thawed (FT) llama semen (untreated control) and FT samples submitted to different centrifugation times (10 and 20 min) through 500 μl of Androcoll-E™ columns (FT-AE).

	**FT semen (control)**	**FT-AE (500 μl) 10 min**	**FT-AE (500 μl) 20 min**
Concentration(×10^6^ sperm/ml)	33.8 ± 10.0^a^	5.1 ± 4.1^b^	7.4 ± 4.5^b^
Oscillatory motility (%)	6.1 ± 6.0^a^	1.0 ± 1.1^b^	1.0 ± 1.6^b^
Progressive motility (%)	3.0 ± 3.8^a^	1.0 ± 1.7^a^	0.7 ± 1.6^a^
Total motility (%)	9.1 ± 5.8^a^	2.0 ± 2.6^b^	1.7 ± 3.2^b^
Viability (%)	17.3 ± 6.8^a^	21.5 ± 9.8^a^	24.7 ± 11.9^a^
HOS+/CB+	22.3 ± 8.9^a^	15.0 ± 8.9^a^	15.8 ± 9.2^a^

*Values are expressed as means ± SD (Experiment 2, n = 10 ejaculates)*.

a,b *For each sperm parameter, different letters between columns indicate significant differences (p < 0.05)*.

None of the sperm pellets resuspended in TALP medium after AE treatment presented thread formation. In both experiments, a significant decrease (*p* < 0.05) in total and oscillatory sperm motility was observed in frozen-thawed samples centrifuged through AE compared to untreated control frozen-thawed semen. However, no significant differences were observed in sperm viability, membrane function, and acrosome integrity (HOS+CB+) between processed and control semen samples (Table 3 for experiment 1 and Table 4 for experiment 2). In addition, no statistical differences were observed in the other HOS/CB categories (HOS+/CB–, HOS–/CB+, and HOS–/CB–) between frozen-thawed samples centrifuged through AE and untreated control frozen-thawed semen (data not shown).

## Discussion

To our knowledge, this is the first study that evaluated the efficiency of a single-layer centrifugation (SLC) through AE to separate frozen-thawed llama sperm from SP and the freezing extender.

A significant decrease in all sperm parameters (total and oscillatory motility, viability, sperm membrane function, and acrosome integrity) was observed after thawing all samples. Several authors have observed the same results, not only in llamas and alpaca (20, 24, 34, 38–40) but also in thawed semen samples from other species [horse: (41); ram: (42, 43); dog: (28); bull: (44); donkey: (29, 30); brown bear: (45)]. The main concern in SACs is the greater loss in sperm quality after thawing (between 65 and 85%) compared to other species, in which the expected decrease in sperm survival is around 50% of the initial value (46). Accordingly, frozen-thawed SAC semen samples harbor a high percentage of damaged and dead cells; therefore, selecting good-quality sperm would be a useful procedure to implement.

Although sperm recovery in frozen-thawed samples processed by AE was low, it was possible to obtain llama sperm free of SP and freezing extender. In contrast, Giuliano et al. (47) observed that the use of AE colloid was unable to separate cooled llama sperm from the egg yolk extender used to preserve the samples. Bertuzzi et al. (32), on the other hand, successfully achieved the separation of llama sperm from SP in raw semen without previous enzymatic treatment using AE, but similarly to the present study, obtained low sperm recovery. In our opinion, it would be possible to obtain greater numbers of recovered sperm by increasing the number of columns used and process the whole ejaculate (and not only an aliquot). In this study, a small volume of sample (100 μl) was placed over the AE column, but considering that the total volume of llama frozen-thawed semen varies between 0.25 and 5 ml, multiple columns could be used to process the whole frozen ejaculate, thus increasing the efficiency of the protocol in terms of sperm recovery. Furthermore, it has been suggested that the initial presence of thread formation in llama ejaculates would seem to play an essential role in the percentage of sperm recovery after the AE treatment (32). These authors observed a lower sperm recovery in samples that showed thread formation compared to the ones that did not. Thus, the same influence could be occurring in llama frozen semen since 40% of thawed samples presented thread formation. In addition, the presence of egg yolk in the diluent could further increase thread formation and exacerbate the situation.

Although total and oscillatory motility decreased in samples processed with AE, sperm viability, membrane function, and acrosome integrity were preserved. The presence of immotile sperm with intact and functional membranes in raw and frozen-thawed llama semen has been reported previously (24, 34, 35, 40). In contrast to our results, Giuliano et al. (47) observed that cooled llama sperm processed with Percoll® maintained the percentages of total sperm motility, while viable sperm decreased in comparison with the untreated cooled semen samples. The differences between the studies could be attributed to the different sperm preservation methods used (freezing vs. cooling) and also to the different colloids applied to process the samples (Androcoll™ vs. Percoll®).

Notably, in the present study and the one by Giuliano et al. (47), the use of AE and Percoll®, respectively, did not accomplish sperm selection, in consequence, the seminal quality of llama cryopreserved samples did not improve. In other species, greater percentages of motile, viable, and morphologically normal sperm with intact acrosomes and DNA have been reported in frozen-thawed samples treated with Androcoll [horse: (25, 26); dog: (27, 28); donkey: (29, 30); brown bear: (31)]. Differences in our results could be due to the fact that these authors used a species-specific Androcoll (Androcoll-E, Androcoll-P, Androcoll-C, and Androcoll-bear). It is worth noting that, currently, there are no reports on the use of a specific type of Androcoll for SACs. In a study performed in dromedary semen, the incorporation of SLC prior to freezing improved post-thaw sperm variables; however, these authors omitted the type of Androcoll used (48). The use of AE in the present study was decided because this treatment proved to be effective as part of a protocol to obtain llama embryos by IVF using raw llama semen previously treated with collagenase and then selected by AE (15). Other differences between the abovementioned studies and ours are the colloid:semen ratio and the centrifugation speed used. Regarding the colloid:semen relation, a similar proportion to the one used in Experiment 2 was tested in stallions (25, 26), while in studies carried out in brown bears, the authors used columns with similar volumes to those used in our experiment 1 (31). On the other hand, in dogs and donkeys, higher volumes of semen were placed over the colloid compared to our study (27–30). Finally, concerning centrifugation velocities, in the species where sperm quality improved after the use of Androcoll columns, lower centrifugation speeds were used (300 *g* 20 min and 600 *g* 10 min) compared to the one applied in the present study (800 *g*). We decided that testing a higher centrifugation speed was a good strategy because, taking into account the rheological characteristics of SAC semen (high structural viscosity and thread formation) and the fact that we did not treat the samples enzymatically prior to centrifugation through Androcoll, we considered that a higher speed could counteract the possible interference of these seminal characteristics with the separation of sperm from SP. Besides, in a previous experiment, we evaluated a lower centrifugation speed (600 *g*) to treat raw llama semen with AE without improving the quality of the sperm obtained and with less sperm recovery compared to 800 *g* (32). These results would seem to indicate that not only is the use of a species-specific Androcoll important when processing the samples but, for the protocol be efficient, the colloid:semen ratio and the centrifugation speed used perhaps also need to be varied according to the species.

Further research needs to be conducted to determine which modifications in the protocol such as colloid density, columns heights, and centrifugation speeds and time are required to improve sperm recovery and to increase the quality of the sperm obtained. Additionally, raw semen samples could be treated with AE before freezing as it has been demonstrated in other species that sperm selection with a SLC prior to cryopreservation can increase post-thaw sperm quality [horse: (41); boar: (49); brown bear: (45)]. One possible disadvantage of AE treatment prior to llama semen cryopreservation could be the low sperm recovery and thus an inadequate sperm pellet for cryopreservation. Added to this, previous studies have shown that freezing llama sperm in the absence of SP does not improve post-thaw cryosurvival (23, 24).

## Conclusions

The use of AE columns, either 500 or 1,000 μl, allowed the separation of cryopreserved llama spermatozoa from SP and freezing extender, preserving sperm viability, membrane function, and acrosome integrity. Of the protocols tested, centrifugation through a 500 μl column of AE at 800 *g* during 20 min would be the method of choice to process frozen-thawed llama semen bound for reproductive biotechnologies such as IVF or intracytoplasmic sperm injection (ICSI). However, different AE:semen ratios, as well as other colloids, should be assayed to improve sperm recovery and to increase the quality of the sperm obtained.

## Data Availability Statement

The raw data supporting the conclusions of this article will be made available by the authors, without undue reservation.

## Ethics Statement

The animal study was reviewed and approved by Committee for the Use and Care of Laboratory Animals (CICUAL) of the Faculty of Veterinary Sciences of the University of Buenos Aires (protocol 2019/22).

## Author Contributions

CG carried out the study and critically read the manuscript. MLB and NV helped collect the samples. FF helped collect the samples and critically read and corrected the manuscript. SG critically read and corrected the manuscript. MVB critically read and translated the manuscript. MC designed and directed the study and wrote the manuscript.

## Conflict of Interest

The authors declare that the research was conducted in the absence of any commercial or financial relationships that could be construed as a potential conflict of interest.

## Abbreviations

SACs: South American camelids
SP: seminal plasma
AE: Androcoll-E™.

## References

[1] Casaretto C Martínez Sarrasague M Giuliano S Rubin de Celis E Gambarotta M Carretero MI. Evaluation of Lama glama semen viscosity with a cone-plate rotational viscometer. Andrologia. (2012) 44:335–41. 10.1111/j.1439-0272.2011.01186.x 21729143

[2] Giuliano S Carretero M Gambarotta M Neild D Trasorras V Pinto M. Improvement of llama (*Lama glama*) seminal characteristics using collagenase. Anim Reprod Sci. (2010) 118:98–102. 10.1016/j.anireprosci.2009.06.005 19616391

[3] Tibary A Vaughan J Reproductive physiology and infertility in male South American camelids: a review and clinical observations. Small Rum Res. (2006) 61:283–98. 10.1016/j.smallrumres.2005.07.018

[4] Fumuso FG Giuliano SM Chaves MG Neild DM Miragaya MH Gambarotta MC. Seminal plasma affects the survival rate and motility pattern of raw llama spermatozoa. Anim Reprod Sci. (2018) 192:99–106. 10.1016/j.anireprosci.2018.02.019 29500052

[5] Ratto MH Gomez C Berland M Adams GP. Effect of ovarian superstimulation on COC collection and maturation in alpacas. Anim Reprod Sci. (2007) 97:246–56. 10.1016/j.anireprosci.2006.02.002 16530992

[6] Condori RL Huanca W Chileno M Cainzo J Valverde F Becerra JJ Effect of follicle-stimulating hormone addition on *in vitro* maturation and cleavage of alpaca (*Vicugna pacos*) embryos. Reprod Fertil Dev. (2010) 23:224 10.1071/RDv23n1Ab252

[7] Huanca W Condori RL Chileno MA Cainzos J Becerra JJ Quintela LA *In vivo* maturation and *in vitro* fertilization of alpaca oocytes. Reprod Fertil Dev. (2010) 23:204 10.1071/RDv23n1Ab211

[8] Berland MA von Baer A Ruiz J Parraguez V Morales P Adams GP. *In vitro* fertilization and development of cumulus oocyte complexes collected by ultrasoundguided follicular aspiration in superstimulated llamas. Theriogenology. (2011) 75:1482–8. 10.1016/j.theriogenology.2010.11.047 21295835

[9] Arqque Monzón DB Influencia del fluido folicular y gonadotropinas en la maduración y fertilización de ovocitos de alpacas huacaya. Puno: Facultad de Medicina Veterinaria y Zootecnia de la Universidad Nacional del Altiplano. (2017). Available online at: http://repositorio.unap.edu.pe/handle/UNAP/8084

[10] Bravo Z Valdivia M Effect of follicular fluid on sperm motility of alpaca *Vicugna pacos* (Linnaeus, 1758). JSM In vitro Fertil. (2017) 2:1013.

[11] Pérez Durand MG Zevallos Aragón JP Perez Guerra UH Comparación de sistemas de cultivo de embriones de alpacas. Rev Investig Altoandin. (2017) 19:157–64. 10.18271/ria.2017.274

[12] Pacompia Torres MH Efecto de Las Gonadotropinas en La Maduración Y Fertilización de Ovocitos En Alpacas (Vicugna pacos). Perú: Universidad Nacional del Altiplano. (2017). Available online: http://repositorio.unap.edu.pe/handle/UNAP/5115

[13] Mamani-Mango G Gonzales MM Hidalgo MR Mendoza Mallma J Ruiz Bejar J Rivas Palma V. Effect of extender and freezing rate on quality parameters and *in vitro* fertilization capacity of alpaca spermatozoa recovered from cauda epididymis. Biopreserv Biobank. (2018) 21:39–45. 10.1089/bio.2018.0021 30256664

[14] Conde P Herrera C Chaves M Giuliano S Director A Trasorras V. *In vitro* production of llama embryos by IVF and ICSI with fresh semen. Anim Reprod Sci. (2008) 109:298–308. 10.1016/j.anireprosci.2007.10.004 18054452

[15] Trasorras V Giuliano S Chaves G Neild D Agüero A Carretero M. *In vitro* embryo production in llamas (*Lama glama*) from *in vivo* matured oocytes with fresh semen processed with Androcoll-E™ using defined embryo culture media. Reprod Dom Anim. (2012) 47:562–7. 10.1111/j.1439-0531.2011.01917.x 21988486

[16] Mortimer D Sperm preparation methods. J Androl. (2000) 21:357–66. 10819443

[17] Henkel R Schill W. Sperm preparation for ART. Reprod Biol Endocrinol. (2003) 1:108. 10.1186/1477-7827-1-108 14617368 PMC293422

[18] Aitken RJ Clarkson JS. Significance of reactive oxygen species and antioxidants in defining the efficacy of sperm preparation techniques. J Androl. (1988) 9:367–76. 10.1002/j.1939-4640.1988.tb01067.x 3215823

[19] Bravo PW Skidmore JA Zhao XX. Reproductive aspects and storage of semen in *Camelidae*. Anim Reprod Sci. (2000) 62:173–93. 10.1016/S0378-4320(00)00158-5 10924824

[20] Aller JF Rebuffi GE Cancino AK Alberio RH Influencia de la criopreservación sobre la movilidad, viabilidad y fertilidad de espermatozoides de llama (*Lama glama*). Arch Zoo. (2003) 52:15–23.

[21] Vaughan J Galloway D Hopkins D. Artificial insemination in alpacas (*Lama pacos*). Kingston, NY: RIRDC Rural Industries Research and Development Corporation (2003).

[22] Fumuso FG Arraztoa CC Chaves MG Neild DM Giuliano SM Miragaya MH. Inseminación artificial de llamas con semen congelado. Res Prelimi Invet. (2018) 20:124. 10.15381/rivep.v28i2.1308030907016

[23] Carretero MI Fumuso FG Chaves MG Miragaya MH Neild DM Cetica P. Comparison of two freeze thawing protocols for llama semen: with and without collagenase and seminal plasma in the medium. Prelim. Res InVet. (2017) 19:57. 27561901 10.1111/and.12691

[24] Fumuso FG Giuliano SM Chaves MG Neild DM Miragaya MH Carretero MI Evaluation of the cryoprotective effect of seminal plasma on llama (*Lama glama*) spermatozoa. Andrologia. (2019) 2019:e13270 10.1111/and.13270 30907016

[25] Macías García B González Fernández L Morrell JM Ortega Ferrusola C Tapia JA Rodriguez Martínez H. Single-layer centrifugation through colloid positively modifies the sperm subpopulation structure of frozen–thawed stallion spermatozoa. Reprod Dom Anim. (2009) 44:523–6. 10.1111/j.1439-0531.2008.01276.x 18992085

[26] Macías García B Morrell JM Ortega-Ferrusola C González-Fernández L Tapia JA Rodríguez-Martínez H. Centrifugation on a single layer of colloid selects improved quality spermatozoa from frozen-thawed stallion semen. Anim Reprod Sci. (2009) 114:193–202. 10.1016/j.anireprosci.2008.08.025 18842364

[27] Dorado J Gálvez MJ Morrell JM Alcaráz L Hidalgo M. Use of single-layer centrifugation with Androcoll-C to enhance sperm quality in frozen-thawed dog semen. Theriogenology. (2013) 80:955–62. 10.1016/j.theriogenology.2013.07.027 23987984

[28] Urbano M Dorado J Ortiz I Morrel JM Demyda-Peyrás S Gálvez MJ Effect or cryopreservation and single layer centrifugation on canine sperm DNA fragmentation assessed by the sperm chromatin dispersión test. Anim Reprod Sci. (2013) 143:118–25. 10.1016/j.anireprosci.2013.10.005 24210910

[29] Ortiz I Dorado J Morrell JM Crespo F Gosálvez J Gálvez MJ. Effect of single-layer centrifugation or washing on frozen-thawed donkey semen quality: Do they have the same effect regardless of the quality of the sample? Theriogenology. (2015) 84:294–300. 10.1016/j.theriogenology.2015.03.021 25917884

[30] Ortiz I Dorado J Morrell JM Diaz-Jimenez MA Pereira B Consuegra C. Comparison of sperm selection techniques in donkeys: motile subpopulations from a practical point of view. Anim Reprod. (2019) 16:282–9. 10.21451/1984-3143-AR2018-0133 33224288 PMC7673585

[31] Anel-Lopez L Ortega-Ferrusola C Álvarez M Borragán S Chamorro C Peña FJ. Improving sperm banking efficiency in endangered species through the use of a sperm selection method in brown bear (*Ursus arctos*) thawed sperm. BMC Vet. Res. (2017) 13:200. 10.1186/s12917-017-1124-2 28651537 PMC5485503

[32] Bertuzzi ML Fumuso FG Giuliano SM Miragaya MH Gallelli MF Carretero MI. New protocol to separate llama sperm without enzymatic treatment using Androcoll-E™. Reprod Domest Anim. (2020) 55:1154–62. 10.1111/rda.13755 32594592

[33] Director A Giuliano S Carretero M Pinto M Trasorras V Miragaya M Electroejaculation and seminal characteristics in llama (*Lama glama*). J Camel Practice Res. (2007) 14:203–6.

[34] Carretero MI Neild D Ferrante A Caldevilla M Arraztoa C Fumuso F. Effect of cryoprotectant and equilibration temperature on *Lama glama* sperm cryopreservation. Andrologia. (2015) 47:685–93. 10.1111/and.12319 25059904

[35] Giuliano S Director A Gambarotta M Trasorras V Miragaya M. Collection method, season and individual variation on seminal characteristics in the llama (*Lama glama*). Anim Reprod Sci. (2008) 104:359–69. 10.1016/j.anireprosci.2007.02.016 17383121

[36] Harrison RA Vickers SE. Use of fluorescent probes to assess membrane integrity in mammalian spermatozoa. J Reprod Fertil. (1990) 88:343–52. 10.1530/jrf.0.0880343 1690300

[37] Carretero MI Pigretti C Bertuzzi ML Fumuso FG Test hipoósmotico combinado a la tinción de coomassie blue en espermatozoides de llama. Spermova. (2018) 8:129–32. 10.18548/aspe/0006.10

[38] Santiani Acosta A Evangelista Vargas S Valdivia Cuya M Risopatrón González J Sánchez Gutierrez R. Effect of the addition of two superoxide dismutase analogues (tempo and tempol) to alpaca semen extender for cryopreservation. Theriogenology. (2013) 79:842–6. 10.1016/j.theriogenology.2012.12.012 23375779

[39] Stuart CC Vaughan JL Kershaw CM de Graaf SP Bathgate R. Effect of diluent type, cryoprotectant concentration, storage method and freeze/thaw rates on the post-thaw quality and fertility of cryopreserved alpaca spermatozoa. Sci Rep. (2019) 9:12826. 10.1038/s41598-019-49203-z 31492923 PMC6731240

[40] Fumuso FG Giuliano SM Chaves G Neild DM Miragaya MH Bertuzzi ML. Incubation of frozen-thawed llama sperm with seminal plasma. Andrologia. (2020) 2020:e13597. 10.1111/and.13597 32352585

[41] Hoogewijs M Morrell J Van Soom A Govaere J Johannisson A Piepers S. Sperm selection using single layer centrifugation prior to cryopreservation can increase thawed sperm quality in stallions. Equine Vet J. (2011) 43:35–41. 10.1111/j.2042-3306.2011.00489.x 22082444

[42] Hernández PJE Fernández RF Rodríguez SJL Juárez RE Soto MYG García RAD Effect of cryopreservation of sheep semen related to its viability and acrosomal status. Rev Salud Anim. (2012) 34:78–83.

[43] Jha PK Shahi Alam MG Mansur AAL Naher N Islam T Bhuiyan MU. Cryopreservation of Bangladeshi ram semen using different diluents and manual freezing techniques. Cryobiology. (2019) 89:35–41. 10.1016/j.cryobiol.2019.06.001 31173735

[44] Sathe S Shipley CF Cryopreservation of semen. In: Hopper RM, editors. Bovine Reproduction. 1st Edition. Iowa: John Wiley & Sons, Inc (2015).

[45] Álvarez-Rodríguez M Álvarez M Anel-López L López-Urueña E Manrique P Borragán S. Effect of colloid (Androcoll-bear, percoll, and puresperm) selection on the freezability of brown bear (*Ursus arctos*) sperm. Theriogenology. (2016) 85:1097–105. 10.1016/j.theriogenology.2015.11.021 26764151

[46] Watson PF. The causes of reduced fertility with cryopreserved semen. Anim Reprod Sci. (2000) 60–1:481–92. 10.1016/S0378-4320(00)00099-3 10844218

[47] Giuliano SM Santa Cruz R Arraztoa CC Fumuso FG Bertuzzi ML Carretero MI Selección espermática de semen refrigerado de llama con diluyente a base de yema de huevo. Spermova. (2019) 9:35–41. 10.18548/aspe/0007.05

[48] Malo C Crichton EG Morrell JM Pukazhenthi BS Johannisson A Splan R. Colloid centrifugation of fresh semen improves post-thaw quality of cryopreserved dromedary camel spermatozoa. Anim Reprod Sci. (2018) 192:28–34. 10.1016/j.anireprosci.2018.02.005 29525206

[49] Martinez-Alborcia MJ Morrell JM Gil MA Barranco I Maside C Alkmin V. Suitability and effectiveness of single layer centrifugation using androcoll-P in the cryopreservation protocol for boar spermatozoa. Anim Reprod Sci. (2013) 140:173–9. 10.1016/j.anireprosci.2013.06.015 23890802

